# Nanotoxicity: a challenge for future medicine

**DOI:** 10.3906/sag-1912-209

**Published:** 2020-06-23

**Authors:** Ramazan AKÇAN, Halit Canberk AYDOGAN, Mahmut Şerif YILDIRIM, Burak TAŞTEKİN, Necdet SAĞLAM

**Affiliations:** 1 Department of Forensic Medicine, Faculty of Medicine, Hacettepe University, Ankara Turkey; 2 Department of Forensic Medicine, Faculty of Medicine, Afyonkarahisar Health Sciences University, Afyonkarahisar Turkey; 3 Department of Nanotechnology and Nanomedicine, Graduate School of Science and Engineering, Hacettepe University, Ankara Turkey

**Keywords:** Nanotechnology, nanotoxicity, bio-nanomaterials, future medicine

## Abstract

**Background/aim:**

Due to nanomaterials’ potential benefits for diagnosis and treatment, they are widely used in medical applications and personal care products. Interaction of nanomaterials, which are very small in size, with tissue, cell and microenvironment, can reveal harmful effects that cannot be created with chemically identical and larger counterparts in biological organisms. In this review, a challenge for future medicine, nanotoxicity of nanomaterials is discussed.

**Materials and methods:**

A detailed review of related literature was performed and evaluated as per medical applications of nanomaterials their toxicity.

**Results and conclusion:**

Most authors state “the only valid technology will be nanotechnology in the next era”; however, there is no consensus on the impact of this technology on humankind, environment and ecological balance. Studies dealing with the toxic effect of nanomaterials on human health have also varied with developing technology. Nanotoxicology studies such as in vivo-like on 3D human organs, cells, advanced genetic studies, and -omic approaches begin to replace conventional methods. Nanotoxicity and adverse effects of nanomaterials in exposed producers, industry workers, and patients make nanomaterials a double-edged sword for future medicine. In order to control and tackle related risks, regulation and legislations should be implemented, and researchers have to conduct joint multidisciplinary studies in various fields of medical sciences, nanotechnology, nanomedicine, and biomedical engineering.

## 1. Introduction

As humanity stepped into the 21st century from the beginning of the 1990s, it began to encounter dozens of new invisible and unknown concepts that would lead to huge growths and developments like nanotechnology. Nanotechnology has become a worldwide billion-dollar industry by producing high-volume, commercial nanomaterials (NMs), including fullerenes, quantum dots (QDs), carbon nanotubes (CNs), and metal-oxide nanoparticles (NPs). Nanotechnology continues to take part in many applications in all areas of human activities (health, food and nutrition, water treatment, production and engineering, etc.) and in our daily life. It is becoming increasingly important due to its beneficial effects on important issues such as energy production, application to technological devices and consumer products to gain new features. Due to its potential benefits for diagnosis and treatment, it has been widely used in healthcare and personal care products. As of 2014, it was reported that 6214 organizations from 32 countries used nanomaterials in 1814 consumer products, most of which (42%) were shown to be in the field of health [1]. The “nanodatabase” is an inventory of commercially marketed products containing nanoparticles designed in the European consumer market, and includes more than 3000 products. According to the data of this inventory, the most usage area is in the health category (close to 2000), more than 900 of them are cosmetics and personal care products. The most widely used nanomaterial for these purposes is silver, followed by titanium and silicon [2]. In parallel with these intense developments in nanotechnology, the issue of whether nanomaterials have toxic effects has begun to come to the agenda. Interaction of nanomaterials, which are very small in size, with tissue, cell and microenvironment, can reveal harmful effects that cannot be created with chemically identical and larger counterparts in biological organisms. Nanotechnology is discussed in this review, as it is an important challenge and a double-edged sword that awaits future medicine.

## 2. Nanotechnology

Nanotechnology is a multidisciplinary field of science, where unique phenomena enable new applications, to design and synthesize products and applications based on the synthesis of nanometer (10-9 meters) molecules. As the particle size decreases at the nanoscale, it is known that the physical properties of the particles can be altered, and features such as resistance, conductivity, durability, lightness, reactivity, longevity and large surface sizes are gained. In this way, it can be used to create new products and applications [3].

## 3. Nanomaterial

### 3.1. Definition

Nanomaterial is a structure that is the size of a virus particle and at least one size (height, width or length) less than 100 nanometers (10−7 meters). They are classified according to their characteristics such as size, dimension (0, 1, 2, 3D), content (carbon-based, inorganic-based, organic-based, composite-based etc.), composition, shape (nanoparticle, nanofiber, nanostick, nanotube etc.), and source (natural, synthetic).

**Figure 1:**
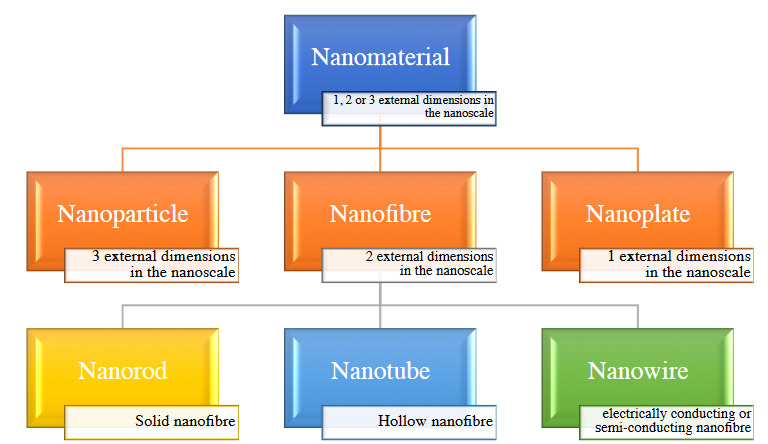
Hierarchy of terms related to nanomaterials.

The most commonly used nanomaterial types are the nanoparticle, the all three dimensions of which are equal to each other and smaller than 100 nanometers, the nanofiber, the two dimensions of which are equal to each other and the other dimension is different from nanosize, and the carbon nanotubes with cylindrical molecules consisting of carbon atoms, as small as 1 nm in diameter and several micrometers in length [1]. Nanoparticles can be classified as organic-inorganic or can be classified in different ways according to different features such as size, molecular structure, and form of production. Hierarchy of terms related to nanomaterials showed in Figure 1.

### 3.2. Composition of nanomaterials

#### 3.2.1. Metal-based

Metal-based NPs are an important class of NPs that are synthesized due to their functions as semiconductors, electroluminescent and thermoelectric materials [4]. These antibacterial NPs have been used in drug delivery systems to reach areas previously unavailable to access by conventional medicine in biomedicine. Recently, interest and development in nanotechnology have been increased, and so many studies have been conducted to evaluate whether the original features of these NPs such as large surface area to volume ratio, negatively affect the environment [5]. Researchers have since determined that various metal and metal-oxide NPs have many hazardous effects on the cells such as oxidation and breakage of DNA, mutations, change of morphology, decreased cell viability, stimulated apoptosis and necrosis, and reduced proliferation [4].

#### 3.2.2. Carbon-based

Typical carbon-based nanomaterial is carbon nanotubes. Carbon nanotubes were first discovered by Iijima and Ichihashi [6] and Bethune et al [7] in 1993. Carbon nanotubes can show significant electrical conductivity [8]. Also, their tensile strength [9] and thermal conductivity [10] are outstanding due to their nanostructure and the strength of the bonds between carbon atoms. Because of these properties of CNs, they can be utilized in many areas of technology from biomedicine to nanoelectronics.

#### 3.2.3. Metal-oxide

Metal-oxide NPs are used as industrial catalysts. TiO2 nanoparticles may disrupt insulin response in Fao cells and cause pregnancy complications in some animal model studies [11, 12]. Studies have showed that other metal-oxide nanoparticles have adverse effects on reproduction and neonatal development [13, 14].

#### 3.2.4. Quantum dots

**Figure 2:**
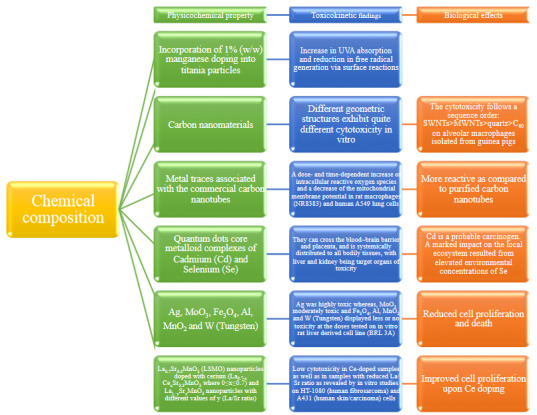
Biological effects due to chemical composition of nanomaterials.

Quantum dots are engineered nanoscale crystals that can transport electrons and they can covert a spectrum of light into different colors. Quantum dots make possible to study cell processes and may notably improve the diagnosis and treatment of diseases such as cancers [15,16]. Some studies showed that QDs have effects on reproductive dysfunction, TH signaling, estrogen receptor activation, and endocrine impairing activity [17–19]. Biological effects due to chemical composition of nanomaterials are summarized in Figure 2 [20–25].

## 4. Nanoparticle

The International Organization for Standardization (ISO) defines the nanoparticle as a nanoobject with all three external dimensions in the nanoscale of about 1 to 100 nm [26, 27]. They can be found naturally in nature, but they are also produced industrially. 

## 5. Nanotoxicity

### 5.1. Definition

Nanotoxicology focuses on determining the adverse effects of nanomaterials on human health and the environment. Nanotoxicology searches for establishing and identifying the harms of engineered nanomaterials and requires a multidisciplinary team approach including toxicology, biology, chemistry, physics, material science, geology, exposure assessment, pharmacokinetics, and medicine [28]. Engineered nanomaterials are used in many fields such as automotive and aerospace (car tires, glass, fuel cells), agriculture (food processing, production, packaging, storage), construction (cement-based material, insulation, exterior, self-cleaning glass and paint, etc.), energy (thermoelectric, solar cells, long-life batteries, fossil fuel, nuclear energy), health and medicine (diagnosis, treatment, regenerative medicine, surgery, implant), information and communication (flat TV screens, electronic devices), security and defense industry (detection, protection, localization, unmanned combat vehicles), textiles (self-cleaning or stain-free products), cosmetics (sunscreens, toothpaste, make-up products), etc. [29]

On the one hand, while it is used in the diagnosis and treatment of diseases in the field of biomedicine, doubts have begun to arise that it may cause diseases. The painful experience of human beings with carcinogenic products such as tobacco products and asbestos, which they initially thought innocent, also caused a question mark for NPs. Because some NPs have long, thin, fibrous structure asbestos-like, show fibrogenic and toxic effects “Can nanoparticles be asbestos of the future?” caused the question to be asked [30].

Factors such as exposure time, dose, aggregation and concentration, particle size and shape, surface area and charge play a key role in the toxicity assessment of nanomaterials [31].

### 5.2. Factors 

#### 5.2.1. Size

**Figure 3:**
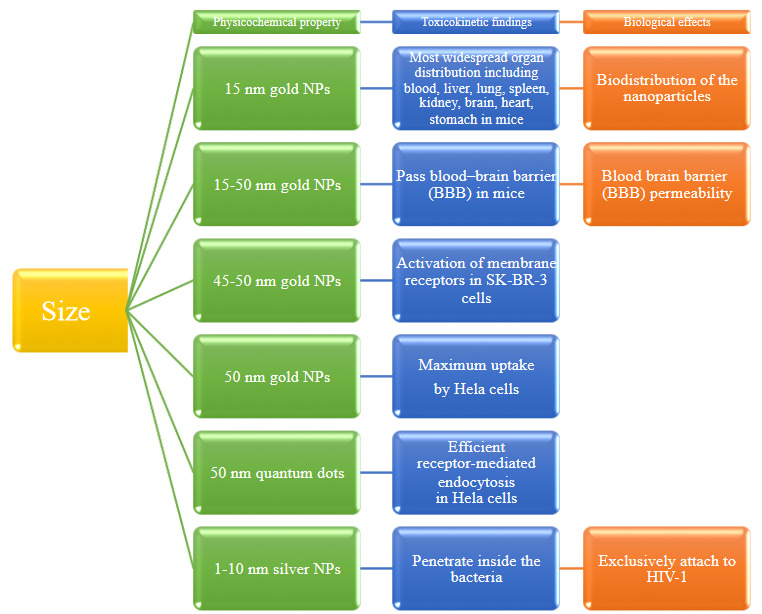
Biological effects due to size of nanomaterials.

There are several ways that size can affect the toxicity of a nanoparticle and showed in Figure 3 [32–37]. For example, the reduction in size of the nanomaterials results an increase in the particle surface area. This causes more molecules to bind to the surface area, so results in an increase in toxic effect [38]. Particles of different sizes can deposit in various places of the lungs and are cleared from the lungs at different rates [39].

#### 5.2.2. Particle surface, surface chemistry and charge

**Figure 4:**
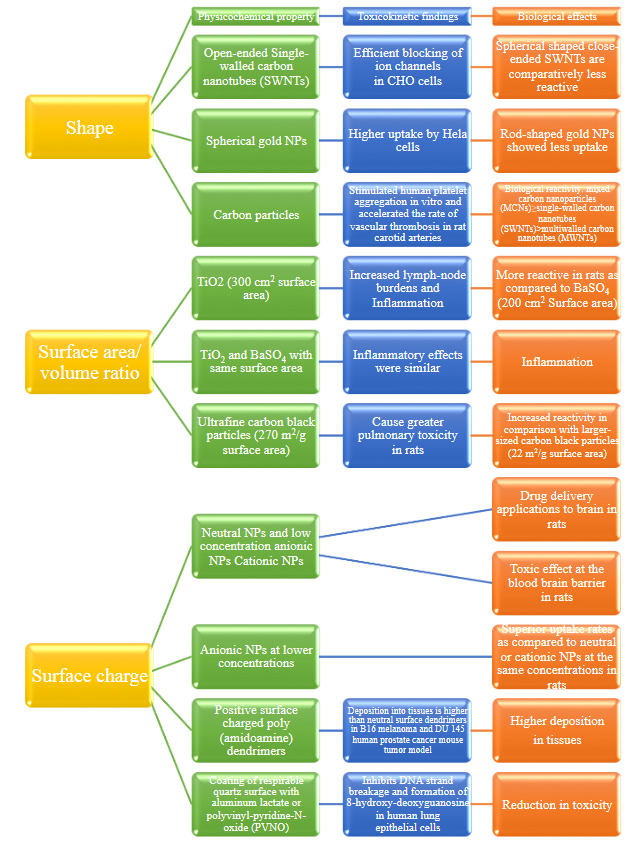
Biological effects due to shape, surface area/volume ratio and charge of nanomaterials.

Extended surface area and fine surface structure of the NMs are properties that help better interaction between microenvironment and nanomaterial biologically. Nanomaterials are covered with coatings and according to their function; they can be positive or negative charged. Electron and atomic force microscopes can be utilized for topographic characterization, so surface chemistry can be evaluated. Studies have showed that these factors can affect the toxicity rate of nanoparticles [40,41]. Biological effectsare showed in figure 4 [32, 42–50].

#### 5.2.3. Dosage

Nanomaterials are known to have dose-dependent toxic effects by inhalation, and there are many publications regarding this issue. Recent studies that the evaluation of mass concentration measurement within the scope of toxicological dosing alone gives false results and does not explain the whole relationship between the nanomaterials and exposed tissue [51].

### 5.3. Exposure routes and ADME

Inhalation is the most common and best-known route among nanomaterial exposure ways. In addition, they can also enter the human body through the skin, digestion or injection.

Nanoparticles are thought to play a role in the development of some diseases by acting on the lungs and other systems with various pathogenic mechanisms. Particles smaller than 0.1 μm can reach distal airways with respiratory units [52]. The inhaled NPs come to the respiratory epithelium and pass through the pores in the alveoli-capillary membrane, first to the interstitium and then to the systemic circulation through blood and lymphatic circulation. In experiments in mice, it has been demonstrated experimentally that NPs applied into the trachea pass into systemic circulation in this way [53].

In studies conducted to reveal the possible toxic effects of NPs on human health, NPs of different character were applied in different ways (inhalation, intratracheal, intravenous, intraperitoneal, etc.) and in different doses, and parameters such as transition to systemic circulation in living organisms, accumulation in tissues, inflammation in tissues, other immune responses and excretion of NPs from the body have been studied. In a study conducted in five healthy volunteers, it has been observed that ultra-fine carbon particles smaller than 100 nm quickly enter the systemic circulation in a short time like 10 minutes after inhalation and maintain their level in the systemic circulation for about an hour [54]. In a study in mice, the 60-day tissue distribution of magnetoelectric NPs of different sizes administered intravenously was investigated by electron microscopy, in approximately one week all NPs reached peak deposition in the lung, but the elimination of large particles of 600nm from the lung was slower than small particles [55]. 

Nanoparticles with short size and spiral structure entering the body are destroyed in tissues by macrophages. However, nanotubes with high aspect ratio reach to the pleura like asbestos fibers and accumulate around the pores there. These fibrous particles cannot be phagocyted, and proinflammatory, genotoxic mitogenic mediators are released by mesothelial cells. Thus, an inflammation and damage process begin [56]. This inflammation that starts in the lungs, on the one hand causes pulmonary endothelial dysfunction and stimulation of pulmonary reflexes, on the other hand activates the platelets and increases the thrombotic activity. In addition, inflammation in the vascular area can cause vascular endothelial dysfunction, causing cardiovascular disorders such as impaired heart rate and rhythm, atherosclerotic plaque formation and rupture [52]. Nanoparticles stimulate both natural and acquired immunity, and causing an inflammatory response. Stimulation of both the macrophage/monocyte, neutrophil, dendritic, natural killer cells responsible for natural immunity and the dendritic cells and lymphocyte responsible for acquired immunity, proinflammatory cytokines, lipid mediators and free radicals are released, resulting in neutrophilic or eosinophilic lung inflammation. Immunomodulatory effects of NPs may differ according to their physicochemical properties such as size, surface structure, electric charge, aggregation ratio [31].

## 6. Entry routes of nanoparticles into the human body

It is inevitable that the human being, who is a social entity, has contact with the nanomaterials around it. A lot of research has been conducted about nanomaterials that have damage different parts of the body. Nanomaterials most often enter the body through the respiratory tract and are in intensive contact with the lungs. The entry of nanomaterials into the body is also very common through skin contact and the gastrointestinal tract. Also, implants and injections allow nanomaterials to enter the body [57]. 

### 6.1. Inhalation exposure

The size of the nanoparticles, its resistance to gravity, and its spreading pattern determine the area in which it will settle in the respiratory tract. Nanoparticles absorbed into the body through the respiratory tract cleaned in different parts of the respiratory system by mucociliary layer and macrophages or they clustered in the lungs and spread to the body with blood circulation [58]. Sajid et al. stated that 33% of the inhaled nanoparticles can be removed from the body by the defensive system of the respiratory tract [59]. Animal studies reported that carbon nanotubes produce fibrosis, inflammation and granuloma in the lungs, and these toxic effects in the lungs cause systemic cardiovascular disorders [60]. Besides, it was stated that the inhaled nanoparticles can reach different organs of the body including the brain, and the evaluation of the risk of association with prostate cancer was investigated [61,62]. 

### 6.2. Dermal exposure

The three effective factors in the absorption of nanoparticles from the skin are the physicochemical properties of nanoparticles, the physicochemical properties of the tool dispersing the penetrating molecule, and the location and skin conditions. Cosmetic cream, lotion and toothpaste are nanoparticle-based tools that are often used in skin exposure. Nanoparticles usually accumulate in the stratum corneum and dermis [63,64]. It is also stated that some of the nanoparticles absorbed from the skin can leak into the bloodstream.

### 6.3. Ingestion

Nanoparticles are effectively absorbed from the gastrointestinal tract directly or through secondary ingestion of inhaled particles. It is important to note that nanoparticles with a high probability of accidental ingestion such as metal compounds and pesticides. This can often be ignored, as it is thought to occur only deliberately or because of gross negligence. Also, poor absorption of nanoparticles from the intestines and metabolism in the liver contributes to this situation [65,66].

## 7. Medical use of nanoparticles

As a relatively new subdivision of medical sciences, nanomedicine takes place among rising disciplines in parallel to the nanotechnological developments. Thanks to the potential of modification of nanoparticle characteristics nanoparticles have a wide range of applications. Therefore, a number of nanoparticles are currently utilized or being studied in certain medical areas such as treatment of diseases or malignancies, surgery, medical implants, smart drug delivery systems, gene delivery, diagnosis/imaging, tissue engineering, regenerative medicine, and antimicrobial resistance and etc. 

### 7.1. Cancer diagnosis and treatment 

The use of nanoparticles in cancer diagnosis, imaging in particular, and treatment increase everyday. In order to reveal tumor sites more accurately quantum dots are utilized with magnetic resonance imaging. On the other hand, cancer biomarkers can be sensed by nanoparticle based test chips such as lab on a chip for noninterventional cancer diagnosis at the earliest stage [67].

Carbon nanotubes are used for revealing mutations in DNA and detection of biomarkers. Dendrimers and nanoparticles can be utilized as contrast agents for imaging, and in mechanisms of smart (targeted or controlled release) drug delivery [68].

Lantanide (Gd3+ and Yb3+) functionalized gold nanoparticles were used in vivo for both imaging (MRI and CT) and for therapeutic (photothermal) purposes. Additionally, ion-doped nanomaterials are used in bio-imaging medical area [69].

### 7.2. Gene therapy

As a widely studied area gene therapy is dedicated to prevention and treatment of genetic disorders by correction of defective genes. This can be performed through delivery or replacement of the repaired or correct gene by several methods. This approach has potential use certain types of cancers, infections, cardiovascular diseases, autoimmune diseases, and monogenic diseases such as hemophilia.

### 7.3. Treatment of neural degeneration 

As in other treatment strategies, treatment of degenerative diseases or posttraumatic pathologies focuses on regeneration and protection of neural tissue, and guided axon growth. Therefore, nanomedical applications are promising in terms of treatment of Parkinson’s, Alzheimer’s diseases, and regeneration of axonal damage. Use of nanoparticles showing high affinity for circulating amyloid-β (Aβ) subtypes potentially suppress symptoms of Alzheimer’s disease [70]. 

### 7.4. Tissue engineering 

This is a commonly known topic by professionals of regenerative medicine, nanomedical and biomedical engineers. It has applications regarding repair or reproduce damaged tissues by various forms and compositions of biocompatible, biodegradable nanomaterial-based bio-scaffolds with minimum side effects.

### 7.5. Antimicrobial activity

A number of metallic nanoparticles are known to show antimicrobial activity, which can be used in combination of medications to reduce antibiotic resistance, as well. Gold, silver, zinc oxide, and etc. nanoparticles take place among such agents. These nanoparticles are also utilized to produce a number of surgical or implantable devices to the body [71].

### 7.6. Orthopedic implants

A number of implants such as bone tissue engineering materials, nanostructured implantable materials, and those produced by surface modification or coating are applied in orthopedic surgeries. Synthetic and natural polymers take place among common nanomaterials used for tissue engineering of bone/cartilage. These are collagen, hyaluronic acid, chitosan, titanium alloys, ceramic-coated metal-oxides (such as alumina, zirconia and titania), hydroxyapatites, and carbon nanomaterials such as graphene or diamond [72].

In order to achieve bioactivity, better mechanical properties and higher osteo-conductivity for faster and more efficient healing process, carbon nanocomposites containing ceramic or polymer matrix are used [73].

### 7.7. Dental application

Nanomaterials applied in dentistry are mostly antimicrobial, therapeutic and reinforcemental materials. They also used for polishing the enamel surface, in dental fillings and in dental implants. Composites are carbon nanotubes, graphene, hydroxyapatite, iron oxide Zirconia, silica-based nanomaterials, titanium and silver nanoparticles [74,75].

### 7.8. Cardiovascular applications

Natural and synthetic nanomaterials are also used in heart tissue bioengineering. For biocompatibility, alginate and collagen are frequently used, while synthetic polyesters such as poly-L-lactic and poly (lactic co-glycolic) acids are commonly used.** **In addition, carbon nanotubes are used for coating stents and coronary implants [76].

### 7.9. Dermal applications

Skin implants that enhance tissue repair process are frequently used in wound healing. Although this frequency varies according to the clinical need, it consists mostly of poly (lactic-co-glycolic acid)/chitin markers that mimic human keratinocytes and fibroblasts as autologous skin grafts [77].

## 8. Toxic effects of nanoparticles on systems

Since nanoparticles enter the body in three main ways, it is known by experimental studies that it causes toxic effects in different systems. This section describes the toxic effects of nanomaterials on systems mostly by experiments on animals.

### 8.1. Circulatory system

Nemmar et al. detected cardiac oxidative stress and DNA damage in a study of intravenous administration of iron oxide nanoparticles in mice [78]. Magaye et al. reported a cardiac toxicity-arrhythmia in the study of intravenous administration of Ni nanoparticles in rats and observed toxic effects in organs such as liver, spleen and lung [79]

### 8.2. Digestive system

Arefian et al. reported that 100 ppm zirconia oxide nanoparticles cause damage to the liver in rats [80]. Also, iron oxide nanoparticles cause liver toxicity in mice [81].

### 8.3. Endocrine system

Yousefi et al. reported that oral form iron oxide nanoparticles cause irregularities in thyroid hormones in rats [82].

### 8.4. Immune system

Xu et al. reported that Ti02 nanoparticles in mice caused a serious increase in the number of white blood cells [83]. Besides, iron oxide nanoparticles cause an increase in the number of white blood cells, and the liver and spleen are the most affected organs immunologically [84]. 

### 8.5. Respiratory system

Cai et al. reported that metal nanoparticles (Cobalt oxide, nickel oxide, titanium oxide) applied by oropharyngeal aspiration cause toxicity in the lungs [85]. Similarly, Sadeghi et al. determined that iron oxide nanoparticles cause lung toxicity in rats [86].

### 8.6. Urinary system

Saranya et al. stated that zinc oxide, iron oxide and copper nanoparticles cause toxic effects on kidney cells in several monkeys, pig and bovine [87]. Besides, Fartkhooni et al. reported that TiO2 nanoparticles injected intraperitoneally cause degeneration in rat kidneys [88].

### 8.7. Nervous system

Studies were carried out on animal ears and eyes related to vision and hearing toxicity, and minimal toxicity was detected or no toxicity was detected generally [89,90].

### 8.8. Reproductive system

Mozaffari et al. determined that zinc oxide nanoparticles injected intraperitoneally in mice caused a decrease and loss in seminiferous tubule cells [91]. Besides, Kong et al. stated that nickel nanoparticles cause a decrease in FSH and LH hormone levels and changes in sperm motility in rats [92].

## 9. Toxicity mechanisms of nanoparticles

**Figure 5:**
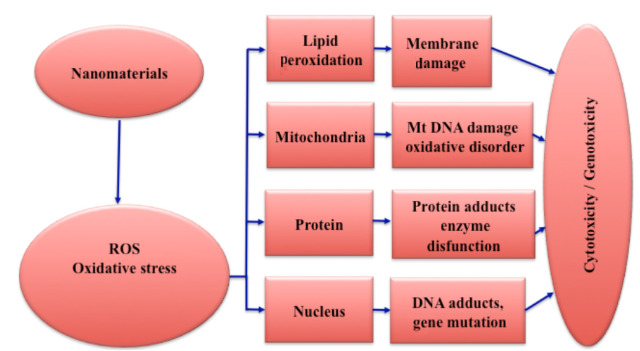
ROS and nanomaterial toxicity

Mechanical effects due to the physicochemical properties of nanoparticles cause toxicity. The basic mechanism of toxic effect formation is reactive oxygen species (ROS) formation, either directly or indirectly. ROS formation is toxic in vitro by multiple mechanisms in the cell [93]. ATP synthesis in mitochondria occurs as a result of the reduction of molecular oxygen to water. During this event superoxide anions and radicals containing different oxygen are formed. ROS formed are known as hydroxyl radical, single oxygen, hydrogen peroxide and superoxide anion radicals [94]. Overproduction of these radicals, which play a role in mitogenic response and cellular signaling and leads to disruption of physiological functions in cells [95,96]. The damage caused by nanomaterials to the cell is cytotoxic and genotoxic (Figure 5). Since nanomaterials have small dimensions, they cause more ROS production due to their specific surface area and high surface reactivity [97]. 

It is revealed in studies in living tissues such as human erythrocytes and skin fibroblasts that different types of nanomaterials cause toxicity by ROS activation [98]. Kim et al. determined that nano-Ag causes oxidative stress and genotoxicity in cultured living tissue also Mei et al. determined that nano-Ag creates mutations by increasing ROS formation in mice [99,100]. Hsin et al. reported that nano-Ag caused cytotoxicity by activating ROS in the mitochondrial pathway [101]. Akhtar et al. reported that silica nanoparticles cause cytotoxicity in the cell membrane and cause cytotoxicity in mouse embryonic fibroblasts through the production of ROS and lipid peroxidation of nano-CuO [102,103]. Girgis et al. proposed that nano-Au caused toxicity by causing an increase in oxidative stress in mice [104]. Shvedova et al. reported that single-walled CNTs cause cytotoxicity in keratinocytes and bronchial epithelial cells leading to ROS production and mitochondrial dysfunction [105]. Winnik and Maysinger determined that quantum dots cause cytotoxicity by increasing ROS production [106]. It is reported that cytotoxic effect of nano-ZnO in human bronchial epithelial cells by increasing ROS production [107]. Nano-FeO was reported to have a cytotoxic effect by increasing ROS formation and apoptosis, also comparing the cytotoxic effects of nano- Ti02, Co3O4, ZnO and CuO in hepatocyte cells, it was found that the most cytotoxic effect was in nano-CuO [108,109]. 

Other factors contribute to the toxicity of nanomaterials, such as surface area, surface coating, molecular size, shape, oxidation status, solubility and degree of aggregation and agglomeration [110]. It is determined that increasing the toxic effect of nanoparticles is directly proportional to the decrease in size. Yoshida et al. reported that amorphous nanosilica causes toxicity in the human cell, both by increasing ROS formation and by damaging DNA [111,112]. Besides, only in the evaluation based on its size, the smaller the nanoparticles, the more toxic it is to the organs [113]. Studies were reported that wire-shaped nanomaterials cause DNA damage and toxic effects through ROS production [114]. Studies were carried out on the effect of the shape of nanomaterials on toxicity, it was reported that the difference in shape does not make a critical difference in the toxic effect of nano Au in human skin keratinocyte cells [115]. On the contrary, a study on nano-ZnO crystals, it was reported that the hexagonal crystals have a more toxic effect than the rod-shaped ones [116]. Biocompatibility and nanoparticle contact area are directly proportional. A study was carried out in zebrafish embryos by Ispas et al. observed that dendritic ones were more toxic than spherical ones [117]. One of the nanomaterials commonly used in drug delivery systems is silica. Nanosilica causes different toxic effects in different pore volumes [118]. Oh et al. reported that the toxicity of the cationic charged nanosilica-titania particles is high [119]. Studies were carried out about size, shape and the relationship of surface parts of quantum dots with nanotoxicity [106,120]. In toxicity studies on fullerenes, the groups bound to the surfaces of these nanomaterials have a determining role in the effect of toxicity. Since it was stated that fullerenes cause cytotoxicity by producing free oxygen radicals, there are also fullerenes with antioxidant activity by adding malonyl groups to their surface [110]. Studies on the effect of nanomaterial solubility on toxicity were conducted. Studer et al. reported that ZnO nanoparticles have a less toxic effect than soluble copper metal [120]. Shen et al. determined that the dissolution of nano-ZnO cells is effective in the important emergence of the cytotoxic effect [121]. Mahto et al. reported that quantum dots dissolve in water, increasing ROS production and causing cytotoxicity [122]. UV and visible light have affected the stabilization of nano-TiO2 and nano-ZnO materials. In this way, photoexcitation through electrons causes toxicity [123]. Studies were carried out on graphene and aggregation toxicity used in many biomedical fields such as drug delivery systems, biosensors and labeling [124]. Also, Kim et al. noted the importance of agglomeration and aggregation in nano-Ag induced toxicity [99]. 

It continues to be researched in different organisms such as rodents, humans and plants in toxicity studies. Multiple areas differ according to the type of nanomaterial carbon and metallic nanomaterials are frequently used in the engineering area. Besides, the use of metal nanomaterials in cosmetics, medicine and food is also a common area.[125]. Sun creams and lotions containing nanotitanium and nanozinc show toxic effects on the skin and the environment depending on the frequency of use [126]. It is shown by the researchers that nanocopper oxide is effective in cytotoxicity and DNA damage, also carbon nanotubes have a toxic effect on cells [127,128]. 

## 10. Toxicity testing

In vitro experiments are performed more frequently than in vivo experiments, and questions about dosing are important in determining toxicity. One of the models used in the toxicity test is in vitro sedimentation diffusion and dosimeter. This model lies in the clear distinction between exposure (concentration in the cell environment), the dose accumulated on the cell surface and the cellular dose. Information about the time to release a given dose allows us to evaluate the dose rate as a determinant of response [129]. 

Since in vitro methods that determine cell viability and proliferation are frequently used in determining toxicity, methods such as gene expression analysis, genotoxicity detection and in vitro hemolysis are also used. Additionally, there are microscopic and spectroscopic methods for the evaluation of physicochemical structure in the cell such as scanning electron microscopy/energy dispersive X-ray spectroscopy (SEM-EDX), transmission electron microscopy (TEM), atomic force microscopy (AFM), video-enhanced differential interference contrast (VEDIC) microscopy and fluorescence spectroscopy. The combined use of all these tests makes it easier to detect nanotoxicity [130]. A concise list to summarize previously conducted studies regarding currently used toxicity tests, the purpose of the tests, and the target nanomaterials is presented in Table [131–147].

**Table:** A summary of literature related toxicity tests of nanomaterials [131-147].

Toxicity test	Purpose	Nanomaterials
Transmission electron microscopy	Determination of intracellular localization	TiO2, silver,fullerene [131–133]
Light microscopy	Physicochemical properties	Singled walled carbon nanotubes, silver [132,134]
Hemoglobin estimation	Hemolysis	SiO2 [135]
Micronucleus test	Genotoxicity	Different types of nanoparticles [136]
Commet assay test	DNA damage	Metal, metal oxide nanoparticles [137]
Lactate dehydrogenase	Cell viability	Carbon nanoparticles [138,139]
Tetrazolium salts		Carbon nanoparticles, fullerenes [140,141]
Alamar Blue		Quantum dots [142]
Propidium iodide		Carbon nanoparticles [143,144]
Neutral red assay test		Carbon nanotubes [140,145]
Caspase-3 activity	Apoptosis	Silver nanoparticles [132]
Acridine orange/ethidium bromide		Silver nanoparticles [146]
ROS production	Oxidative stress	TiO2 [131]
Levels of glutathione peroxidase, catalase, superoxide dismutase		Polymeric nanoparticles [147]
Lipid peroxidation, vitamin E		Singled walled carbon nanotubes [105]

Exposure to nanoparticles through the respiratory tract often causes adverse effects on the lung. There are many studies on determining the detection of lung toxicity, and organ-on-a-chip studies have been important in recent years. Zhang et al. evaluated nanotoxicity by better imitating human responses with the chip in a 3D human lung model similar to in vivo. Also, this study showed the importance of organ-based toxicity with realistic models [148]. Studies in mouse placenta determined that nanoparticles pass through the placenta and show a toxic effect. Yin et al. reported that chip and TiO2 nanoparticle exposure-related studies in the 3D human placenta model might have similar toxic effects [149]. Besides, nanotoxicity studies were carried out with the integration of a cell-on-a-chip (CoC) with a microfluidic system [150]. 

## 11. Concerns, future aspects and concluding remarks

Studies utilizing nanotechnology have been continuing rapidly in the last twenty years, which boosts related investment, industrial activities, marketing, and economic planning. This results in increase of number of related good and bad actors in the area. Each actor takes the heed of different priorities that might be controversial to others. Medical professionals considers biocompatibility, biodegradability and effectiveness of nanomaterials as priority, while professionals interested in industrial activities, marketing, and economic issues may prioritize scaling up production of new devices or nanomaterials, and decreasing costs and timescales. This inconsistency also raises questions about nanomaterials’ potential adverse effects. Since most authors state that “the only valid technology will be nanotechnology in the next era”, there is no consensus on the impact of this technology on humankind, environment and ecological balance. Following increasing regulatory demands regarding use of nanomaterial-based medical devices and advanced therapeutic medicinal products; governments have installed certain institutional projects. Out of projects investigating nanomaterials’ safety, the National Cancer Institute in United States points out that “most engineered nanoparticles are far less toxic than household cleaning products, insecticides used on family pets, and personal care products”. Similarly, European Union installed BIORIMA (BIOmaterial Risk MAnagement) project that aims developing an integrated risk management framework for the safe handling of nano-biomaterials used in medical applications, and to assess and manage certain factors potentially arising from manufacturing and use of such materials.

Studies dealing with the toxic effect of nanomaterials on human health have also varied with developing technology. Nanotoxicology studies such as in vivo-like on 3D human organs, cells also advanced genetic studies are beginning to replace conventional in vitro analytical methods [151,152]. In vitro testing methods might require assessment of multiple challenging steps such as physicochemical properties of nanomaterials, the environment-target cell, cellular uptake and epigenetic interaction [153]. Omic approaches; next generation sequencing, transcriptomics and proteomics, have provided considerably more information regarding the toxicity of the complex cellular processes triggered by interaction of nanomaterials with the microenvironment [154,155]. Also, an important point is personalized toxicology. Possible genetic susceptibility to toxicity of nanomaterials should also be carefully studied under this topic [156]. The analysis of data obtained through novel technological developments and nanotoxicological studies is getting more and more difficult. In respect of above discussed issues, extraordinary increase of use of nanomaterial-based medical agents and devices come up with a challenge for future medicine. Nanotoxicity and adverse effects of nanomaterials in exposed producers, industry workers, and patients make nanomaterials a double-edged sword for future medicine. In order to control and tackle related risks, regulation and legislations should be implemented, and researchers have to conduct joint multidisciplinary studies in various fields of medical sciences, nanotechnology, nanomedicine, and biomedical engineering.

## Acknowledgement/Disclaimers/Conflict of interest

No funds were received in support of this work. This study was presented in Taiwan-Turkey Science Summit that was held in Ankara, Turkey, on 1–4 April 2018. There is no conflict of interest between the authors concerning the materials or methods used in this study or the findings specified in this paper.

## Informed consent

There is no need informed consent about this work.

This study was presented at the Taiwan-Turkey Science Summit entitled “Translation of Cells, Nanomaterials and Signaling Molecules into Regenerative Medicine” between April 1 to 3, 2018.
